# Shared correlates of maternal and childhood overweight in Cameroon: a cross-sectional analysis of demographic and health survey data

**DOI:** 10.1186/s12889-023-16164-y

**Published:** 2023-06-29

**Authors:** Lambed Tatah, Luchuo Engelbert Bain, Eugene Kongnyuy, Felix Assah, Jean Claude Mbanya

**Affiliations:** 1 Health of Populations in Transition Research Group (HoPiT), University of Yaoundé I, 8046 Yaoundé, Cameroon; 2 Medical Research Council Epidemiology Unit, University of Cambridge, CB2 0QQ Cambridge, UK; 3 Department of Psychology, Faculty of Humanities, University of Johannesburg, Johannesburg, South Africa; 4 International Development Research Centre, IDRC, Ottawa, ON Canada; 5 UNFPA, Kinshasa, Democratic Republic of Congo

**Keywords:** Obesity, Overweight, Cameroon, Mother and child

## Abstract

**Background:**

Overweight parents are likelier to bear overweight babies, who are likelier to grow into overweight adults. Understanding the shared risks of being overweight between the mother-child dyad is essential for targeted life course interventions. In this study, we aimed to identify such risk factors in Cameroon.

**Methods:**

We conducted secondary data analysis using Cameroon’s 2018 Demographic and Health Surveys. We used weighted multilevel binary logistic regressions to examine individual, household, and community correlates of maternal (15–49 years) and child (under five years) overweight.

**Results:**

We retained 4511 complete records for childhood and 4644 for maternal analysis. We found that 37% [95%CI:36–38%] of mothers and 12% [95%CI:11–13%] of children were overweight or obese. Many environmental and sociodemographic factors were positively associated with maternal overweight, namely urban residence, wealthier households, higher education, parity and being a Christian. Childhood overweight was positively associated with a child being older and a mother being overweight, a worker, or a Christian. Therefore, only religion affected both mothers overweight (aOR: 0.71[95%CI:0.56–0.91]) and childhood overweight (aOR 0.67[95%CI: 0.5–0.91]). Most of the potentially shared factors only indirectly affected childhood overweight through maternal overweight.

**Conclusion:**

Besides religion, which affects both mothers and childhood overweight (with the Muslim faith being protective), much of childhood overweight is not directly explained by many of the observed determinants of maternal overweight. These determinants are likely to influence childhood overweight indirectly through maternal overweight. Extending this analysis to include unobserved correlates such as physical activity, dietary, and genetic characteristics would produce a more comprehensive picture of shared mother-child overweight correlates.

## Background

Overweight and obesity are serious global health problems. These conditions pose a particular challenge in low- and middle-income countries (LMICs), where obesity is rising rapidly and worsening the burden of diabetes, hypertension and other non-communicable diseases (NCDs) [1]. A recent analysis of 24 African countries showed that the rising prevalence of women’s overweight and obesity significantly affected all the 24 study countries, with Egypt reporting the highest levels of overweight (44%) and obesity (39%) in 2014 and Ghana almost doubling its overweight and tripling its obesity prevalences between 1993 and 2014 [2]. Literature on body weight profiles in LMICs reports higher overweight prevalence in people who are older, married, highly educated, watch television at least once a week, and reside in urban areas [3]. Children in urban areas, who are younger than six months, are males or have overweight mothers are also likelier to be overweight and obese [4–6]. Notwithstanding these overarching insights, up-to-date analyses tracking overweight and obesity prevalence and their determinants are still lacking in many LMICs [7].

Cameroon, a sub-Saharan African LMIC with just over 26 million people, falls in the category of countries with sparse literature in this area. Initial data shows a growing burden of overweight and obesity [8–10], making a case for further exploration of the country’s weight profiles. A recent cross-sectional survey of adults in a town (Baham) in the west region of the country showed that 31.1% and 18.9% of 485 respondents were overweight and obese, respectively [8]. These numbers are slightly comparable to a two-decade-old cross-sectional study in Yaoundé, the country’s political capital, which reported that one in every two women was overweight and 1 in 5 was obese [9]. A more extensive study of the two largest Cameroonian cities in 2018 reported that over 32% of women aged 15–49 were obese [10]. Compared to adults, the prevalence of overweight in children has been lower. It ranges from 8% according to an analysis of 4518 children aged between 6 and 59 months in the 2011 demographic and health surveys data [11] to nearly double according to cross-sectional studies from Douala (Cameroon’s economic capital) and the North West Region [12–14]. Like other LMICs, studies show that older women, women with higher levels of education and those who consume sweetened beverages have higher odds of being obese in Cameroon [8, 9]. In children, maternal obesity and overweight, high birth weight, age, and being from the grass field regions are associated with childhood overweight [5–8], while being Muslim was the only reported protective factor [11, 13]. Albeit no consensus, these sparse data indicate a growing problem in the country that needs further studies.

Childhood overweight is a known risk factor for adulthood overweight and obesity. Overweight mothers are also more likely to develop gestational diabetes [5], increasing the risk of overweight in their babies [11]. Understanding the burden and shared risk factors in these population groups is essential for public health planning and targeted interventions from a life course perspective. Researchers have used the widely available demographic survey data to examine the determinants of obesity and overweight in women, men, or children separately. There is a lack of studies that compare these determinants across population subgroups. The rapidly changing lifestyle is worsening the risk of obesity among children and women, signalling an urgent need to reverse the trend. We aimed to explore the 2018 Cameroon Demographic and Health Surveys to identify the correlates of overweight shared by mothers and children in Cameroon. Specifically, we determine overweight correlates in mothers and children and track those shared by both population groups.

## Methodology

### Study design setting and participants

We analysed the 2018 Cameroon Demographic and Health Surveys (DHS) to determine the correlates of overweight shared by mothers and children in Cameroon. We reported the study following the “Strengthening the Reporting of Observational Studies in Epidemiology (STROBE)” guidelines [15], with emphasis on elements of a cross-sectional study.

The study area was Cameroon, a lower-middle-income central African country with just over 26 million people. Like most sub-Saharan African countries, Cameroon is rapidly urbanising and exposing many people to sedentary living. Its diet is also transitioning from traditional fibrous diets to obesogenic ones high in fats, sugars and salts [16]. The country’s health indicators are generally poor as its system has been underfunded, fragile, and under constant pressure from infectious diseases and maternal and child health challenges for decades. NCDs are now rising faster and increasing the challenge of preserving health. Moreover, policies for NCD prevention are lagging, and current programmes still prioritise mother and child health [17].

Because data on mother and child health were more readily available, we focused our study on these populations. Our study participants were children aged 0–59 months and their mothers aged 15–49. These age brackets correspond to those used by the DHS programme in surveying population health and demographics across multiple countries. Children’s inclusion criteria for the survey were any child in the sampled households within the age range. We included all children with anthropometric measurements but excluded twins below seven months. We excluded this subset because twins are likely to have smaller weights, especially in the early period of life (first six months) when they heavily rely on exclusive breastfeeding. This cut-off was relatively arbitrary as child feeding practices in Camerron are diverse [18], and there was no information regarding the average time for twins’ weight to catch up with singletons in the country. Mothers’ inclusion criteria for the survey were all mothers in the 15–49 age group in the surveyed households. We included all mothers with anthropometric measurements but excluded all pregnant women from our analysis.

### Data source

The data source for this analysis was the children’s dataset of the 2018 Cameroon DHS. The DHS is a nationally representative quinquennial survey conducted in over 85 low- and middle-income countries [19]. The programme uses structured questionnaires to collect household and individual data on health indicators such as maternal and child health [20]. The DHS uses a multi-stage sampling process to select clusters, households and individuals within country sub-divisions, ensuring national, regional and urban/rural representativeness. Details on the sampling are published elsewhere [19]. This analysis used the children’s data file containing sociodemographic and biometric data on children and their mothers. We included only the complete cases for the outcome variables of interest. As with every secondary data analysis, the sample size was capped by the original survey; however, the sizeable multi-indicator survey implies that available samples are likely to exceed the minimum required sample size for our analysis.

### Variables

The outcome variable was “overweight”, a binary variable denoting whether or not a participant was overweight. This variable was based on the survey recorded mothers’ body mass index (BMI) (measured in kg/m^2^) and children’s weight-for-height (WfH) (calculated as the number of standard deviations from the median WfH value). Participants were assigned “Yes” if BMI ≥ 25 or if WfH > 2 and “No” if they did not meet these criteria. For descriptive purposes only, we defined another categorical variable, “nutrition status”, to capture more details of individual weight profiles: “Underweight”, “Normal weight”, “Overweight”, and “Obese”. Mothers were considered “Underweight” if their BMI < 18.5, “Normal weight” if 18.5 ≤ BMI < 25, “Overweight” if 25 ≤ BMI < 30, and “Obese” if their BMI ≥ 30. Children were considered “Underweight” if their WfH < 2, “Normal weight” if -2 ≤ WfH ≤ 2, “Overweight” if 2 < WfH ≤ 3, and “Obese” if WfH > 3 [21].

We included 16 explanatory variables selected because of their known association with the outcome variables [22] and their availability in the DHS dataset. We grouped the variables into six household/community-level and ten individual-level variables.

The household/community-level variables were [1] “Household size”, defined as the number of individuals living in the household. Household size was categorised as “≤2”, “3–5”, or “≥6” people based on the counts of household residents; [2] “House head’s gender”, defined as the sex of the household head (female or male); [3] “House head’s age”, defined as the age of the household head and categorised into five groups: “15–24”, “25–34”, “35–44”, “45–54”, and “55+”; [4] “Residence,” indicated whether a household was located in an urban or rural setting; [5] “Wealth index”, defined as the wealth quintile of the household and was labelled as poorest, poorer, middle, richer, or richest; and [6] “Responsible for household purchases”, defined as the person who decided on larger household purchases. It could be the “Mother alone”, “Mother and someone”, and “Partner or someone else”.

Individual variables were the following: [1] “Mother’s age”, grouped as 15–24, 25–34, and 35–49 years; [2] “Child’s age”, grouped as 0–6 months, 7–12 months, 1–2 years, and 3–5 years; [3] “Mother’s age at first birth”, defined as the age at which mother gave first birth and categorised in three groups similar to “Mother’s age”; [4] “Child’s sex”; [5] “Mother’s religion”, coded as “Christianity”, “Islam”, “African religion and others”; [6] “Mother’s education” coded as “none”, “primary”, “secondary”, and “higher”; [7] “Mother’s occupation” coded as “Working” or “Not working”; [8] “Mother’s marital status” coded as “never married”, “previously married”, and “lives with partner”; [9] “Mother’s parity”, defined as the number of times the woman has been pregnant and coded as “1”, “2”, “3”, and “4 or more”; [10] “Mother exposed to mass media” coded as “Yes” indicating exposure to mass media and “No” indicating no exposure. The media exposure variable was created from three other variables that measured the frequency of watching television, reading newspapers or magazines, and listening to the radio. Women who responded that they never engaged in these activities were categorised as not exposed to mass media, while those who responded with a frequency of less than once a week, at least once a week, or almost every day were grouped as exposed to mass media.

We simply excluded the records with missing data points for the outcome variable. To ensure that we retained as many records for the descriptive analysis as possible, we split the dataset into child and mother files before removing missing data. Consequently, the number of records in each file was different, and there were records without matches in each file, which were later excluded during the modelling process.

### Statistical analysis

We conducted all analyses in the R statistical software (version 4.02). Firstly, we used the Pearson chi-squared tests to explore the relationship between the predictor and outcome variables in bivariate analyses. Secondly, we used a multilevel binary logistic regression to examine the correlates of maternal and child overweight. We built four models (Models 0-III) for each outcome variable. Model 0 evaluated the variance in overweight attributed to the clustering at the primary sampling units (PSUs) and households; Model I contained the household/community-level variables; Model II contained the individual-level variables; Model III included all the individual and household/community-level variables. We used both Akaike’s Information Criterion (AIC) and pseudo r-squared (pseudo R^2^) to compare model fitness, selecting the model with the least AIC and highest pseudo R^2^ as the best-fitted model. We used the Variance Inflation Factor (VIF) to test for multicollinearity. The minimum, maximum, and mean VIF were 1.00, 3.74, and 1.74, respectively, indicating no evidence of high collinearity among the studied variables. We used women’s sample weights (v005/1,000,000) and R’s “survey” package to weight our descriptive statistics. We also adjusted for the complex sampling structure of the data using the “glmer” package with the weight argument for our weighted multilevel regression. We presented the outputs of the regression analyses using adjusted odds ratio (aOR) with their respective 95% confidence intervals (CIs). Statistical significance was set at p < 0.05.

### Bias

The DHS incorporates rigorous mechanisms to reduce bias in survey data, including systematic multi-stage sampling, a large enough sample size for important indicators, using well-trained researchers to administer questionnaires and transparent data processing and sharing steps. We further reduced bias by excluding pregnant women and twin children younger than seven months from the analysis and applying the survey weights to account for the complex sampling techniques.

### Ethical considerations

We did not seek an additional ethical clearance since the DHS datasets are publicly available with guidelines for ethical use. The DHS reports ethical approvals from the Ethics Committee of ORC Macro Inc. and the Ethics Boards of partner organisations of various countries, including the Health Ministries. The DHS follows the required standards to protect respondents’ privacy. ICF International ensures that the survey complies with the US Department of Health and Human Services’ regulations regarding respect for human subjects. We complied with DHS recommendation for ethical data usage, and further information about the data usage and ethical standards are available at http://goo.gl/ny8T6X.

## Results

### Characteristics of study participants

From the initial 9733 mother-child pair observations in the children’s file, we retained 4531 complete unweighted records for childhood and 4644 complete unweighted records for maternal overweight analysis. Table 1 summarises the sociodemographic characteristics of the mothers and children, stratified by weight status (weighted estimates). More than half of the households were in rural areas, and over two-thirds of households had at least six members. About half of the mothers and children were between 25 and 34 and 3–5 years, respectively. Over a third of mothers (37% [95% CI: 36–38%]) and 12% (95% CI: 11–13%) of children were overweight or obese. Overweight mothers were likelier to have overweight children and vice versa.

**Table 1:** Bivariable analysis of predictors of overweight among mothers and children in Cameroon (weighted estimates)

Characteristic	Mothers			Children		
	All mothers	Overweight mothers	p-value	All children	Overweight children	p-value
n (%)	4756.5	1751.9 (37.1)		4724.6	570.7 (12.1)	
**Household size, n (%)**			0.184			0.474
≤2	62.2 (1.3)	33.2 (1.9)		18.5 ( 0.4)	3.6 (0.6)	
3–5	1324.0 (27.8)	486.0 (27.7)		1352.1 (28.6)	149.0 (26.1)	
≥6	3370.2 (70.9)	1232.7 (70.4)		3354.0 (71.0)	418.1 (73.3)	
House head’s gender, female, n (%)	852.4 (17.9)	365.4 (20.9)	0.012	774.5 (16.4)	90.3 (15.8)	0.777
**House head’s age, n (%)**			< 0.001			0.115
15–24	93.8 (2.0)	19.5 (1.1)		100.1 ( 2.1)	8.0 (1.4)	
25–34	1278.0 (26.9)	404.6 (23.1)		1302.9 (27.6)	144.8 (25.4)	
35–44	1489.8 (31.3)	654.6 (37.4)		1557.1 (33.0)	176.7 (31.0)	
45–54	919.9 (19.3)	318.9 (18.2)		861.5 (18.2)	138.1 (24.2)	
55+	975.1 (20.5)	354.3 (20.2)		903.0 (19.1)	103.1 (18.1)	
**Wealth index, n (%)**			< 0.001			0.594
Poorest	973.3 (20.5)	146.3 (8.4)		1013.7 (21.5)	114.9 (20.1)	
Poorer	1079.4 (22.7)	296.5 (16.9)		1075.0 (22.8)	135.9 (23.8)	
Middle	996.0 (20.9)	379.5 (21.7)		994.3 (21.0)	103.1 (18.1)	
Richer	835.5 (17.6)	420.8 (24.0)		807.6 (17.1)	102.0 (17.9)	
Richest	872.3 (18.3)	508.7 (29.0)		834.1 (17.7)	114.8 (20.1)	
Residence, urban, n (%)	2164.2 (45.5)	1110.9 (63.4)	< 0.001	2106.0 (44.6)	236.2 (41.4)	0.415
**Responsible for household purchases, n (%)**			< 0.001			0.479
Mother alone	390.4 (8.2)	185.6 (10.6)		388.6 ( 8.2)	48.0 (8.4)	
Mother and someone	1652.5 (34.7)	746.2 (42.6)		1669.1 (35.3)	183.8 (32.2)	
Partner or someone else	2713.6 (57.1)	820.1 (46.8)		2667.0 (56.4)	338.9 (59.4)	
Child’s sex, girl, n (%)	2303.4 (48.4)	836.5 (47.7)	0.596	2299.2 (48.7)	261.0 (45.7)	0.169
**Child’s age, n (%)**			0.019			< 0.001
0–6 months	644.1 (15.1)	246.2 (15.5)		603.8 (12.8)	111.2 (19.5)	
6–12 months	509.8 (11.9)	163.2 (10.3)		519.7 (11.0)	78.2 (13.7)	
1–2 years	845.1 (19.8)	292.1 (18.3)		978.6 (20.7)	97.3 (17.0)	
3–5 years	2278.2 (53.3)	890.7 (55.9)		2622.6 (55.5)	284.0 (49.8)	
**Mother’s age, n (%)**			< 0.001			0.464
15–24	1379.3 (29.0)	343.0 (19.6)		1345.9 (28.5)	154.2 (27.0)	
25–34	2393.3 (50.3)	946.9 (54.0)		2420.6 (51.2)	286.8 (50.3)	
35–44	983.8 (20.7)	462.0 (26.4)		958.2 (20.3)	129.7 (22.7)	
**Mother’s parity, n (%)**			0.435			0.594
1	686.0 (14.4)	225.5 (12.9)		672.7 (14.2)	90.3 (15.8)	
2	884.9 (18.6)	320.2 (18.3)		868.6 (18.4)	112.0 (19.6)	
3	821.0 (17.3)	316.3 (18.1)		838.1 (17.7)	94.3 (16.5)	
4+	2364.6 (49.7)	889.9 (50.8)		2345.2 (49.6)	274.1 (48.0)	
**Mother’s age at first birth, n (%)**			< 0.001			0.035
15–24	4202.6 (88.4)	1441.7 (82.3)		4193.5 (88.8)	488.1 (85.5)	
25–34	537.1 (11.3)	297.1 (17.0)		516.2 (10.9)	81.6 (14.3)	
35–44	16.8 (0.4)	13.1 (0.7)		14.9 ( 0.3)	0.9 (0.2)	
**Mother’s education, n (%)**			< 0.001			0.793
None	1233.0 (25.9)	151.8 (8.7)		1278.3 (27.1)	147.4 (25.8)	
Primary	1563.3 (32.9)	603.4 (34.4)		1557.5 (33.0)	184.8 (32.4)	
Secondary	1701.3 (35.8)	815.0 (46.5)		1635.0 (34.6)	200.6 (35.2)	
Tertiary	258.9 (5.4)	181.8 (10.4)		253.8 ( 5.4)	37.9 (6.6)	
Mother’s occupation, working, n (%)	3282.5 (69.0)	1180.8 (67.4)	0.273	3265.6 (69.1)	357.0 (62.6)	0.005
**Mother’s marital status, n (%)**			0.014			0.689
Never married	479.4 (10.1)	214.4 (12.2)		406.3 ( 8.6)	55.8 (9.8)	
Previously Married	367.2 (7.7)	122.0 (7.0)		305.0 ( 6.5)	36.4 (6.4)	
Currently lives with partner	3909.8 (82.2)	1415.4 (80.8)		4013.3 (84.9)	478.6 (83.9)	
**Mother’s religion, n (%)**			< 0.001			0.046
Christianity	3261.8 (68.6)	1398.0 (79.8)		3191.1 (67.5)	422.3 (74.0)	
Islam	1326.1 (27.9)	316.3 (18.1)		1353.5 (28.6)	127.9 (22.4)	
African religion and others	168.5 (3.5)	37.6 ( 2.1)		180.0 ( 3.8)	20.5 (3.6)	
**Mother’s media exposure, Yes, n (%)**	2590.8 (54.5)	1340.2 (76.5)	< 0.001	2530.2 (53.6)	316.0 (55.4)	0.649
**Mother’s nutrition status, n (%)**			< 0.001			0.001
Underweight	293.3 (6.2)	0.0 (0.0)		272.5 ( 6.5)	3.4 (0.7)	
Normal weight	2711.3 (57.0)	0.0 (0.0)		2360.5 (56.5)	267.3 (51.9)	
Overweight	1125.6 (23.7)	1125.6 (64.2)		1002.8 (24.0)	160.7 (31.2)	
Obese	626.3 (13.2)	626.3 (35.8)		544.7 (13.0)	83.2 (16.2)	
**Child’s nutrition status, n (%)**			0.001			< 0.001
Underweight	212.9 (5.1)	48.5 (3.1)		223.0 ( 4.7)	0.0 (0.0)	
Normal weight	3475.8 (82.7)	1257.1 (81.1)		3931.0 (83.2)	0.0 (0.0)	
Overweight	328.8 (7.8)	172.2 (11.1)		365.1 ( 7.7)	365.1 (64.0)	
Obese	185.8 (4.4)	71.7 (4.6)		205.6 ( 4.4)	205.6 (36.0)	

Example of table interpretation for the “Child’s nutrition status, n (%)” variable. In the mothers’ column (file), 212.9 (5.1%), 3475.8 (82.7%), 328.8 (7.8%), 185.8 (4.4%) of all mothers have children who are underweight, normal weight, overweight, obese, respectively, while 48.5 (3.1), 1257.1 (81.1), 172.2 (11.1), 71.7 (4.6) of overweight mothers have children who are underweight, normal weight, overweight, obese, respectively. In the children’s column (file), 223.0 ( 4.7), 3931.0 (83.2), 365.1 ( 7.7), 205.6 ( 4.4) of all children are underweight, normal weight, overweight, obese, respectively, while 0.0 (0.0), 0.0 (0.0), 365.1 (64.0), 205.6 (36.0) of overweight children are underweight, normal weight, overweight, obese, respectively

Table 1 Bivariable analyses of predictors of overweight among mothers and children in Cameroon (weighted estimates).

### Correlates of maternal and child overweight

Table 2 shows the potential correlates of maternal and child overweight. Based on the pseudo R^2^, the model fitness for maternal overweight was better than child overweight, indicating that the available covariates were more useful in explaining variation in maternal overweight than child overweight.

**Table 2:** Mixed effect analysis of determinants of overweight among mothers and children in Cameroon

	Mothers				Children			
Characteristic (reference)	Model 0	Model 1 aOR[95%CI]	Model 2 aOR[95%CI]	Model 3 aOR[95%CI]	Model 0	Model 1 aOR[95%CI]	Model 2 aOR[95%CI]	Model 3 aOR[95%CI]
Fixed effects								
Household size (≤ 2 people)								
3–5		0.47[0.24,0.91]*		0.54[0.27,1.07]		0.59[0.15,2.31]		0.37[0.09,1.65]
≥6		0.67[0.35,1.3]		0.63[0.31,1.24]		0.6[0.15,2.36]		0.38[0.09,1.68]
House head’s gender, female (male)		1.22[0.99,1.51]		1.09[0.88,1.36]		1.1[0.84,1.44]		0.92[0.67,1.25]
House head’s age (15–24 years)								
25–34		1.77[0.89,3.5]		1.09[0.54,2.19]		1.55[0.7,3.43]		1.38[0.58,3.27]
35–44		2.69[1.35,5.34]*		1.27[0.63,2.57]		1.53[0.69,3.39]		1.34[0.56,3.23]
45–54		2.17[1.08,4.37]*		1.19[0.58,2.43]		1.62[0.71,3.66]		1.68[0.69,4.09]
55+		1.95[0.97,3.92]		1.18[0.58,2.41]		1.46[0.65,3.31]		1.25[0.51,3.06]
Residence, urban (rural)		4.91[3.8,6.35]*		4.18[3.11,5.63]*		0.93[0.71,1.22]		0.79[0.56,1.11]
Wealth Index (poorest)								
Poorer		2.48[1.86,3.32]*		2.45[1.8,3.33]*		1.33[0.95,1.86]		1.19[0.86,1.66]
Middle		4.01[2.96,5.44]*		3.96[2.86,5.5]*		1.13[0.79,1.61]		1[0.69,1.45]
Richer		7.75[5.65,10.64]*		6.97[4.92,9.86]*		1.18[0.82,1.69]		0.97[0.66,1.44]
Richest		9.4[6.81,12.98]*		8.39[5.77,12.21]*		1.15[0.79,1.66]		1.08[0.7,1.67]
Responsible for purchases (Mother alone)								
Mother and someone		0.9[0.67,1.22]		0.94[0.69,1.27]		0.69[0.47,1]		0.8[0.54,1.19]
Partner or someone else		0.61[0.46,0.83]*		0.83[0.61,1.13]		0.74[0.51,1.07]		0.85[0.57,1.27]
Child’s sex, girl (boy)							0.86[0.7,1.05]	0.86[0.7,1.05]
Child’s age (0–6 months)								
6–12 months							0.87[0.6,1.25]	0.86[0.6,1.24]
1–2 years							0.51[0.36,0.71]*	0.49[0.35,0.69]*
3–5 years							0.58[0.44,0.77]*	0.58[0.44,0.77]*
Mother’s age (15–24 years)								
25–34			2.14[1.71,2.67]*	1.68[1.33,2.12]*			1.09[0.81,1.46]	1.08[0.8,1.46]
35–44			3.31[2.44,4.5]*	2.4[1.74,3.31]*			1.38[0.93,2.06]	1.33[0.88,2.01]
Mother’s education (none)								
Primary			2.87[2.17,3.79]*	1.96[1.48,2.61]*			1.16[0.85,1.59]	1.2[0.87,1.67]
Secondary			3.29[2.42,4.47]*	1.64[1.19,2.27]*			1.11[0.78,1.58]	1.17[0.8,1.71]
Tertiary			5.03[3.2,7.93]*	1.64[1.01,2.67]*			1.33[0.77,2.29]	1.44[0.79,2.62]
Mother’s occupation, working (Not working)			0.93[0.77,1.11]*	1[0.83,1.21]			0.67[0.53,0.84]*	0.66[0.52,0.84]*
Mother’s religion (Christianity)								
Islam			0.89[0.7,1.13]	0.71[0.56,0.91]*			0.7[0.52,0.93]*	0.67[0.5,0.91]*
African religion and others			0.56[0.34,0.91]*	0.59[0.36,0.97]*			1[0.58,1.71]	0.95[0.55,1.64]
Mother’s media exposure, Yes (No)			2.8*[2.25,3.5]*	1.42[1.13,1.78]*			1.01[0.78,1.32]	1.01[0.78,1.32]
Mother’s parity (1)								
2			1.18[0.89,1.56]	1.24[0.93,1.65]			1.04[0.73,1.48]	1.05[0.73,1.5]
3			1.62[1.2,2.19]*	1.68[1.23,2.29]*			1[0.68,1.47]	1.02[0.69,1.5]
4+			1.65[1.22,2.24]*	1.94[1.4,2.68]*			0.97[0.66,1.43]	0.94[0.63,1.41]
Mother’s age at first birth (15-24years)								
25–34			1.26[0.95,1.66]	1.31[0.98,1.74]			1.12[0.8,1.58]	1.15[0.81,1.63]
35–44			4.36[1.1,17.23]*	3.25[0.8,13.16]			0.2[0.01,3.23]	0.16[0.01,2.79]
Mother’s overweight, Yes (No)							1.8[1.41,2.31]*	1.88[1.46,2.42]*
**Random effects**								
PSU standard deviation	1.34	0.88	0.94	0.86	0.85	0.85	0.84	0.84
ICC	0.35	0.19	0.21	0.19	0.18	0.18	0.18	0.18
Model information and fitness								
Number of clusters	428	428	428	428	428	428	428	428
Number of observations	4644	4644	4644	4644	4531	4531	4531	4531
AIC	5003.9	4624.7	4659.0	4469.6	3151.4	3169.8	3052.5	3068.9
Pseudo-R² (fixed effects)	0	0.23	0.19	0.31	0	0.01	0.03	0.04
Pseudo-R² (total)	0.35	0.38	0.36	0.43	0.19	0.19	0.21	0.21

Exponentiated coefficients, 95% confidence intervals in brackets, aOR adjusted Odds Ratios, CI Confidence Interval, * p < 0.05, PSU Primary Sampling Unit, ICC Intra-Class Correlation, AIC Akaike’s Information Criterion

Both mother and child null models did not converge when the household identification was modelled as a complementary or independent random variable. Thus, both null models only included the cluster level for random effects. The null model for maternal overweight showed an intra-class correlation coefficient (ICC) of 0.35, indicating that 35% of the total variation in the odds of maternal overweight was attributable to differences between the clusters. The coefficient was lower for children overweight (0.18). There was a decrease in the ICC of the null model when fixed factors were added to the mothers’ evaluation, suggesting that the proportion of variance in maternal overweight that was due to between-cluster differences decreased as more factors were added. In contrast, the ICC of child overweight remained unchanged as fixed factors were added to the model.

Multiple factors were positively associated with maternal overweight: urban residence, living in wealthier households, being older, higher education, exposure to mass media, and higher parity, while having no religion or any religion other than Christianity was negatively associated with overweight. For children, having an overweight mother was positively associated with being overweight, while being older, having a working mother, or having no religion other than Christianity were negatively associated with being overweight. Therefore, residing in urban areas was associated with a higher likelihood of being overweight in mothers (aOR = 4.18, 95%CI [3.11, 5.63]) but not in children. Mothers from wealthier households were more likely to be overweight than those from the poorest households. Specifically, the odds of being overweight were higher for mothers in the middle (aOR = 3.96, 95%CI [2.86, 5.5]), richer (aOR = 6.97, 95%CI [4.92, 9.86]), and richest (aOR = 8.39, 95%CI [5.77, 12.21]) households, as compared to the poorest. In contrast, household wealth was not associated with being overweight in children. The odds of being overweight were higher for mothers with three (aOR = 1.68, 95%CI [1.23, 2.29]) and four or more children (aOR = 1.94, 95%CI [1.4, 2.68]) as compared to those with one child, but parity was not associated with overweight in children. Regarding a child’s age, the study found that children aged 1–2 years (aOR = 0.49, 95%CI [0.35, 0.69]) and 3–5 years (aOR = 0.58, 95%CI [0.44, 0.77]) had a lower likelihood of overweight as compared to children aged 0–6 months, but child’s age was not associated with overweight in mothers. Children whose mothers were overweight had a higher likelihood of being overweight (aOR = 1.88, 95%CI [1.46, 2.42]) than those whose mothers were not overweight. Mothers working had a lower likelihood of being overweight in children (aOR = 0.66, 95%CI [0.52, 0.84]) compared to those who were not working. However, maternal working status was not associated with being overweight in mothers. Only religion was jointly associated with maternal overweight (aOR: 0.71*[0.56,0.91]) and child overweight (aOR 0.67*[0.5,0.91]), and the associations were in the same direction.

Table 2 Mixed effect analysis of determinants of overweight among mothers and children in Cameroon.

## Discussion

### Main findings

In this secondary data analysis, we tracked the correlates of overweight shared by mothers and children in Cameroon. The study adds to just a few that have separately explored mother and child overweight but offers results that inform overweight control in both mothers and children. We found that many known environmental and sociodemographic factors were positively associated with maternal overweight, including urban residence, higher household wealth, higher education, parity and being a Christian. On the other hand, childhood overweight was positively correlated only with older children, overweight in mothers, mothers’ occupation, and mothers being Christians. Therefore, apart from religion which affected mother and childhood overweight, there was no other factor jointly affecting maternal childhood overweight. However, most factors affecting mothers’ overweight affected childhood overweight indirectly through maternal overweight.

### Study strengths and limitations

The strength of this study relies on the use of a recent nationally representative dataset, which was collected through standardised procedures. We also used appropriate multilevel regression techniques to isolate risk factors. However, the study has limitations that should be considered when interpreting the results. We assessed only the overweight correlates available in the DHS dataset. These were not comprehensive because the survey was not designed to evaluate overweight risk factors. Other potential unobserved correlates, such as those related to physical activity, food environment and diet, and genetics, could improve our model fit and provide a complete picture of overweight correlates in the population. Secondly, our data and analysis were limited to children and women to the exclusion of adolescents and men, who are also important in understanding the NCD risk profile in the population. The third area of concern is that our analysis was limited to one country, highlighting an important risk factor that might be very context-specific and needs multiple context analyses for a comprehensive list of correlates. Finally, we used BMI to measure obesity which has known limitations for measuring individual risks of obesity; however, it has acceptable validity for population-level risks.

### Interpretation of findings

The finding that over a third of mothers in Cameroon were overweight or obese is concerning, as this indicates a high prevalence and underscores the urgent need for comprehensive and targeted interventions to address the growing burden of overweight and obesity in Cameroon, both among mothers and children. This finding is consistent with previous research in sub-Saharan Africa that has shown an increase in the prevalence of overweight and obesity among women of reproductive age, ranging from 15% (Ghana) to 36% (South Africa) [23]. Similar to adults, children are also affected by the overweight problem. The 12% of under-five overweight and obesity observed in Cameroon falls within the range reported for Sub-Saharan Africa: 8% (Cameroon) to 16% (South Africa) [24].

Sociodemographic predictors of overweight among mothers and children are different. Only religion affected both groups, with being Muslim having a protective effect. There is no clear evidence in the literature to suggest that Muslims are less likely to be overweight compared to Christians or other religions, but possible explanations for this association could include dietary restrictions, physical activity patterns, and cultural attitudes towards body weight [25]. For instance, the Islamic religion has specific dietary restrictions, such as abstaining from consuming pork and alcohol and eating healthily [26], which may contribute to a lower risk of obesity and overweight. Certain cultural practices among Muslim populations, such as daily prayers that involve physical movements, may also promote physical activity and thus contribute to a lower risk of obesity and overweight. One religious practice directly associated with weight profile is fasting. Its practice, especially by healthy individuals or those with cardiovascular disease, generally shows favourable associations with lower cholesterol and weight status [27]. Still, the effects of such practices on children are unclear, and evidence suggests that individuals revert to their pre-fasting weight within one month after the fasting period [28, 29]. A further look at the data showed that underweight was highly prevalent in the Muslim group than in other religions. This information may suggest a general undernutrition problem in the Muslim group, leading to fewer people crossing into the overweight band. While there are other possible explanations for the weight profile differences by religion, it is important to note that this relationship is complex and likely influenced by many factors beyond religion alone and those considered here. Future research should investigate the mechanisms underlying the possible association between religion and being overweight. Nonetheless, this finding indicates that religion must be considered when designing NCD control programs that address overweight mothers and children since, in some cases, the focus will be on addressing undernutrition. Moreover, religion helps illustrate the double burden of malnutrition in the country.

We know that the Muslim religion is dominant in Northern Cameroon, where the prevalence of underweight is higher [10] compared to Southern Cameroon where overweight and obesity prevalences are higher [10]. The northern population is largely made up of the Fulani/Fulbe/Bororos, who are physically active as nomads, and their diets have much milk and fewer carbohydrates compared to Southern Cameroon, which could explain the strong but possible confounding correlation between overweight and religion. This geographical variation is supported by the cluster-level variation (ICC) of 19% and 18% in mothers and children, respectively, albeit at a micro-level. This finding suggests that factors operating at the individual level, such as religion, diet, and cultural attitudes, as discussed earlier, may also exert an aggregate influence at the community level. It is conceivable that elements or ideologies associated with communal livelihood contribute to this observed phenomenon [30]. Furthermore, alternative explanations warrant consideration, including community-level factors like food security, exposure to media and nutrition education, disparities in healthcare accessibility, and ethnic attributes. Exploring these factors in greater depth through further research will provide a comprehensive understanding of the intricate interplay between individual and community-level determinants in the context of overweight among women and children.

Given the high prevalence and burdens associated with childhood overweight and obesity, the current findings prompt careful exploration of other mechanisms linked to childhood overweight. Genetic or biological mechanisms could provide alternative explanations [31, 32], but the question remains to what extent in this population. Exploring other study designs that integrate life course approaches in providing plausible explanations could be helpful. For example, our analysis did not consider the birth weight of children. It would have been essential to see how birth weight predicts overweight with time. This reinforces the need for a life course or trends analysis to understand these factors. Population cohorts are rare in Cameroon. Establishing such cohorts can complement well-designed and representative cross-sectional studies in holistically understanding how socioeconomic and biological factors interact to predispose or protect one from becoming overweight. Additionally, older children had lesser prevalence overweight compared with younger children. Literature on child’s age and overweight/obesity studies is conflicting, with studies reporting higher overweight prevalence in older children [33] and others reporting the reverse [34]. The conflicting observation has been attributed to possible measurement error or rapid crossing of growth channels by infants [34].

Correlates of maternal overweight in the present study are consistent with findings from other studies. Women with medium education and wealth are the most susceptible to weight gain [35]. Daran and Levasseur [35] noted that the burden of overweight or obesity in sub-Saharan Africa tends to shift from the most educated women to women with intermediate levels of education. They observed a similar shift in the female BMI distribution with respect to household wealth indicators. These shifts are consistent with the nutrition transition theory [36, 37], in which the burden of obesity moves from the most privileged individuals to the least privileged individuals but first transits through intermediate social groups, in parallel with economic development and the rise of obesity. The social shift described in previous studies reveals the urgent need to adapt health programs and anti-obesity actions in a context where both hunger and overweight coexist and affect specific social groups differently. An urban residence is inextricably linked to higher education and wealth, but the urban environment has other challenges that fuel overweight. Urban areas often have greater availability of high-calorie foods and fewer opportunities for physical activity [38]. Several studies have indicated that parity is a risk factor in maternal overweight and obesity [39–41]. A study of Danish women showed a 7.2% increase in overweight and obesity and a 16.4% increase in obesity alone for pregnant women with higher parity [42]. One possible explanation is the physiological changes that occur during pregnancy and childbirth. Pregnancy leads to weight gain, and the body undergoes hormonal changes that can affect metabolism and fat storage. With each successive pregnancy, these changes may accumulate, making it more challenging for women to lose the extra weight gained during pregnancy.

Finally, the findings highlight the significant impact of a mother’s overweight status and working status on the development of overweight in children. Maternal overweight status has been identified as a critical risk factor for childhood overweight and obesity [43], and the association between maternal working status and overweight is consistent with evidence suggesting that working mothers may have less time and resources to prepare healthy meals and engage in physical activity with their children [44].

### Implications of findings

Our study not only highlights the usefulness of DHS data in monitoring and evaluating NCD risk factors in LMICs but also underscores the need for the DHS programme to extend and routinise anthropometric measures in men to improve the assessment of the country’s NCD risk profiles. By including men in anthropometric measurements, we can gain a more comprehensive understanding of the prevalence and distribution of overweight and obesity and their risk factors, enabling more targeted interventions.

Our study also highlights the heightened risk of overweight in Christian communities to both mothers and children. This finding emphasises the need for tailored interventions that consider the community type in preventing overweight and its associated health consequences. To effectively reduce the burden of overweight and obesity, it is crucial for policymakers and public health officials to consider implementing evidence-based interventions that address the specific needs of mothers and children in various settings, including urban and rural areas, different socioeconomic backgrounds, and different stages of family development.

By implementing such interventions, we can target the complexity and diversity of factors influencing overweight prevalence, ultimately improving the health outcomes of mothers and children. This includes addressing barriers to healthy eating and physical activity, promoting nutrition education and awareness, ensuring equitable access to healthcare services, and fostering supportive environments that encourage healthy lifestyle choices.

## Conclusion

This paper identified religion as the only overweight correlate shared by mothers and children in Cameroon, with the Muslim faith associated with protective effects in both groups. While multiple other social, economic and demographic factors, such as age, household wealth, education, and parity, showed a significant association with maternal overweight, the only other factors associated with child overweight were the child’s age, maternal occupation, and maternal overweight status. Therefore, most variation in childhood overweight is not directly explained by many of the observed determinants of maternal overweight but indirectly through maternal overweight. Including unobserved factors such as physical activity, dietary, and genetic characteristics would produce a more comprehensive picture of overweight risk profiles and shared correlates in mothers and children. Additionally, including fathers’ risk profiles would also improve the image, and we recommend the systematic anthropometric measurements of men in DHS studies. Extending this analysis to similar contexts would show specificity and expose unexpected findings.

## Data Availability

Data are available upon request from the DHS portal https://dhsprogram.com/data/.

## References

[1] Mbanya JC, Assah FK, Saji J, Atanga EN (2014). Obesity and type 2 diabetes in Sub-Sahara Africa. Curr Diab Rep.

[2] Amugsi DA, Dimbuene ZT, Mberu B, Muthuri S, Ezeh AC (2017). Prevalence and time trends in overweight and obesity among urban women: an analysis of demographic and health surveys data from 24 african countries, 1991–2014. BMJ Open.

[3] Tekalegn Y (2021). Determinants of overweight or obesity among men aged 20–59 years: a case-control study based on the 2016 ethiopian demographic and Health Survey. J Obes.

[4] NCD Risk Factor Collaboration (NCD-RisC) (2019). Rising rural body-mass index is the main driver of the global obesity epidemic in adults. Nature.

[5] Sserwanja Q, Mutisya LM, Olal E, Musaba MW, Mukunya D (2021). Factors associated with childhood overweight and obesity in Uganda: a national survey. BMC Public Health.

[6] Weldearegay HG, Gebrehiwot TG, Abrha MW, Mulugeta A (2019). Overweight and obesity among children under five in Ethiopia: further analysis of 2016 national demographic health survey: a case control study. BMC Res Notes.

[7] NCD Risk Factor Collaboration (NCD-RisC) (2017). Worldwide trends in body-mass index, underweight, overweight, and obesity from 1975 to 2016: a pooled analysis of 2416 population-based measurement studies in 128·9 million children, adolescents, and adults. Lancet.

[8] Simo LP, Agbor VN, Temgoua FZ, Fozeu LCF, Bonghaseh DT, Mbonda AGN (2021). Prevalence and factors associated with overweight and obesity in selected health areas in a rural health district in Cameroon: a cross-sectional analysis. BMC Public Health.

[9] Pasquet P, Temgoua LS, Melaman-Sego F, Froment A, Rikong-Adié H (2003). Prevalence of overweight and obesity for urban adults in Cameroon. Ann Hum Biol.

[10] Engle-Stone R, Nankap M, Ndjebayi AO, Friedman A, Tarini A, Brown KH (2018). Prevalence and predictors of overweight and obesity among cameroonian women in a national survey and relationships with waist circumference and inflammation in Yaoundé and Douala. Matern Child Nutr.

[11] Tchoubi S, Sobngwi-Tambekou J, Noubiap JJN, Asangbeh SL, Nkoum BA, Sobngwi E (2015). Prevalence and risk factors of overweight and obesity among children aged 6–59 months in Cameroon: a Multistage, Stratified Cluster Sampling Nationwide Survey. PLoS ONE.

[12] Choukem S-P, Kamdeu-Chedeu J, Leary SD, Mboue-Djieka Y, Nebongo DN, Akazong C (2017). Overweight and obesity in children aged 3–13 years in urban Cameroon: a cross-sectional study of prevalence and association with socio-economic status. BMC Obes.

[13] Navti LK, Ferrari U, Tange E, Parhofer KG, Pozza SB-D (2015). Height-obesity relationship in school children in Sub-Saharan Africa: results of a cross-sectional study in Cameroon. BMC Res Notes.

[14] Wamba PCF, Enyong Oben J, Cianflone K (2013). Prevalence of overweight, obesity, and thinness in Cameroon urban children and adolescents. J Obes.

[15] Cuschieri S (2019). The STROBE guidelines. Saudi J Anaesth.

[16] Global Nutrition Report. Country Nutrition Profiles, Cameroon. 2023. https://globalnutritionreport.org/resources/nutrition-profiles/africa/middle-africa/cameroon/. Accessed 15 Feb 2023.

[17] Tatah L, Mapa-Tassou C, Shung-King M, Oni T, Woodcock J, Weimann A (2021). Analysis of Cameroon’s sectoral policies on physical activity for noncommunicable Disease Prevention. Int J Environ Res Public Health.

[18] Asoba GN, Sumbele IUN, Anchang-Kimbi JK, Metuge S, Teh RN (2019). Influence of infant feeding practices on the occurrence of malnutrition, malaria and anaemia in children ≤ 5 years in the Mount Cameroon area: a cross sectional study. PLoS ONE.

[19] Corsi DJ, Neuman M, Finlay JE, Subramanian SV (2012). Demographic and health surveys: a profile. Int J Epidemiol.

[20] Aliaga A, Ren R. Cluster Optimal Sample Size for Demographic and Health Surveys. 2006.

[21] World Health Organization. Noncommunicable diseases: Childhood overweight and obesity. 2020. https://www.who.int/news-room/questions-and-answers/item/noncommunicable-diseases-childhood-overweight-and-obesity. Accessed 15 Feb 2023.

[22] Pradeilles R, Irache A, Norris T, Chitekwe S, Laillou A, Baye K. Magnitude, trends and drivers of the coexistence of maternal overweight/obesity and childhood undernutrition in Ethiopia: Evidence from Demographic and Health Surveys (2005–2016). Maternal & Child Nutrition. n/a n/a:e13372. 10.1111/mcn.13372 PMC11258774 35615766

[23] Owobi OU, Okonji OC, Nzoputam CI, Ekholuenetale M (2022). Country-Level Variations in overweight and obesity among Reproductive-Aged women in Sub-Saharan Countries. Women.

[24] Danquah FI, Ansu-Mensah M, Bawontuo V, Yeboah M, Kuupiel D (2020). Prevalence, incidence, and trends of childhood overweight/obesity in Sub-Saharan Africa: a systematic scoping review. Archives of Public Health.

[25] Wilhelm L, Hartmann AS, Becker JC, Kisi M, Waldorf M, Vocks S (2019). Thin media images decrease women’s body satisfaction: comparisons between veiled Muslim Women, Christian Women and Atheist Women regarding trait and state body image. Front Psychol.

[26] Iftikhar R. Obesity and Lifestyle Recommendations in the Light of Islam.

[27] Oman D (2018). Public health nutrition, religion, and spirituality. Why religion and spirituality matter for public health: evidence, implications, and resources.

[28] Sadeghirad B, Motaghipisheh S, Kolahdooz F, Zahedi MJ, Haghdoost AA (2014). Islamic fasting and weight loss: a systematic review and meta-analysis. Public Health Nutr.

[29] Hajek P, Myers K, Dhanji A-R, West O, McRobbie H (2012). Weight change during and after Ramadan fasting. J Public Health.

[30] Suglia SF, Shelton RC, Hsiao A, Wang YC, Rundle A, Link BG (2016). Why the Neighborhood Social Environment is critical in obesity Prevention. J Urban Health.

[31] Loos RJF, Yeo GSH (2022). The genetics of obesity: from discovery to biology. Nat Rev Genet.

[32] Bouchard C (2009). Childhood obesity: are genetic differences involved?. Am J Clin Nutr.

[33] Skinner AC, Perrin EM, Skelton JA (2016). Prevalence of obesity and severe obesity in US children, 1999–2014. Obesity.

[34] Ricardo LIC, Gatica-Domínguez G, Crochemore-Silva I, Neves PAR, dos Santos Vaz J, Barros AJD (2021). Age patterns in overweight and wasting prevalence of under 5-year-old children from low- and middle-income countries. Int J Obes.

[35] Daran B, Levasseur P (2022). Is overweight still a problem of rich in sub-saharan Africa? Insights based on female-oriented demographic and health surveys. World Dev Perspect.

[36] Monteiro CA, Moura EC, Conde WL, Popkin BM (2004). Socioeconomic status and obesity in adult populations of developing countries: a review. Bull World Health Organ.

[37] Popkin BM (1993). Nutritional patterns and transitions. Popul Dev Rev.

[38] Gaiha R, Jha R, Kulkarni VS, Obesity. Affluence and Urbanisation in India. 2011.

[39] TAGHDIR M, ALIMOHAMADI Y, SEPANDI M, REZAIANZADEH A, ABBASZADEH S, MAHMUD FM (2020). Association between parity and obesity: a cross sectional study on 6,447 iranian females. J Prev Med Hyg.

[40] Boudet-Berquier J, Salanave B, Desenclos J-C, Castetbon K (2017). Sociodemographic factors and pregnancy outcomes associated with prepregnancy obesity: effect modification of parity in the nationwide Epifane birth-cohort. BMC Pregnancy Childbirth.

[41] Huayanay-Espinoza CA. Parity and Overweight/Obesity in peruvian women. Prev Chronic Dis. 2017;14. 10.5888/pcd14.160282 PMC5662294 29072986

[42] Iversen DS, Kesmodel US, Ovesen PG (2018). Associations between parity and maternal BMI in a population-based cohort study. Acta Obstet Gynecol Scand.

[43] Heslehurst N, Vieira R, Akhter Z, Bailey H, Slack E, Ngongalah L (2019). The association between maternal body mass index and child obesity: a systematic review and meta-analysis. PLoS Med.

[44] Lou Y, Zhu Y, You Q, Jiang Q, Meng X, Di H (2022). Maternal long working hours and offspring’s weight-related outcomes: a systematic review and meta-analysis. Obes Rev.

